# Dense GeV electron–positron pairs generated by lasers in near-critical-density plasmas

**DOI:** 10.1038/ncomms13686

**Published:** 2016-12-14

**Authors:** Xing-Long Zhu, Tong-Pu Yu, Zheng-Ming Sheng, Yan Yin, Ion Cristian Edmond Turcu, Alexander Pukhov

**Affiliations:** 1 College of Science, National University of Defense Technology, Changsha 410073, China; 2 Collaborative Innovation Center of IFSA (CICIFSA), Key Laboratory for Laser Plasmas (MoE) and Department of Physics and Astronomy, Shanghai Jiao Tong University, Shanghai 200240, China; 3 SUPA, Department of Physics, University of Strathclyde, Glasgow G4 0NG, UK; 4 School of Electronic Science and Engineering, Nanjing University, Nanjing 210023, China; 5 National Institute for Physics and Nuclear Engineering, ELI-NP, Str Reactorului, nr. 30, P.O.Box MG-6, Bucharest-Magurele 077125, Romania; 6 Central Laser Facility, STFC Rutherford Appleton Laboratory, Didcot, Oxfordshire OX11 0QX, UK; 7 Institut für Theoretische Physik I, Heinrich-Heine-Universität Düsseldorf, 40225 Düsseldorf, Germany

## Abstract

Pair production can be triggered by high-intensity lasers via the Breit–Wheeler process. However, the straightforward laser–laser colliding for copious numbers of pair creation requires light intensities several orders of magnitude higher than possible with the ongoing laser facilities. Despite the numerous proposed approaches, creating high-energy-density pair plasmas in laboratories is still challenging. Here we present an all-optical scheme for overdense pair production by two counter-propagating lasers irradiating near-critical-density plasmas at only ∼10^22^ W cm^−2^. In this scheme, bright γ-rays are generated by radiation-trapped electrons oscillating in the laser fields. The dense γ-photons then collide with the focused counter-propagating lasers to initiate the multi-photon Breit–Wheeler process. Particle-in-cell simulations indicate that one may generate a high-yield (1.05 × 10^11^) overdense (4 × 10^22^ cm^−3^) GeV positron beam using 10 PW scale lasers. Such a bright pair source has many practical applications and could be basis for future compact high-luminosity electron–positron colliders.

## Abstract (web summary)

High power lasers can produce electron-positron pairs at GeV energies, but doing so through laser–laser collisions would require exceedingly high intensities. Here the authors present an all-optical scheme for pair production by irradiating near-critical-density plasmas with two counter-propagating lasers.

Pair production is one of the fundamental quantum electrodynamics (QED) effects, which is potentially interesting for a variety of applications^1^,^2^,^3^, such as fundamental nuclear and particle physics, laboratory astrophysics and plasma physics, radiography for material science and medical applications. For example, GeV and even TeV positron beams are required for studying highly energetic astrophysical phenomena in laboratories and realizing electron–positron (*e*^−^*e*^*+*^) collider for high-energy particle physics^3^,^4^. Schwinger has predicted the critical electric field^5^ *E*_s_≈1.32 × 10^18^ V m^−1^ for spontaneous creation of pairs out of vacuum by a laser beam. This field corresponds to a light intensity roughly 10^29^ W cm^−2^, which is seven orders of magnitude higher than attainable in current laboratories^4^. It has also predicted that pairs can be produced via the Trident and Bethe–Heitler (BH) processes^6^,^7^ from lasers interaction with high-Z targets. So far, the major way of producing positrons with lasers in experiments relies upon the BH process, which is based on the decay of bremsstrahlung γ-rays from electrons in high-Z targets. It is shown that energetic positrons could be obtained by direct laser–solid interactions^8^,^9^,^10^ or by laser-driven electrons colliding with solid targets^11^,^12^,^13^,^14^. However, the positrons obtained have a low density of ∼10^16−17^ cm^−3^ with a laser energy conversion efficiency to positrons around ∼0.02% only^15^,^16^. There is a need to significantly enhance the positron yield, density and energy, as well as the laser energy conversion for the aforementioned applications.

Under extremely high laser intensities, the laser–matter interaction enters the near-QED regime and the following two critical processes are involved: (1) high-energy photons emission by relativistic electrons quivering in ultraintense laser fields^17^ (*e*^−^+*n*γ_laser_→γ_photon_+*e*^−^, where γ_laser_ represents a laser photon); and (2) pairs creation by real photon–photon annihilation, that is, the multi-photon Breit–Wheeler (BW) process^18^ (γ_photon_+*m*γ_laser_→*e*^−^+*e*^+^). The first process is essentially the nonlinear Compton scattering of laser photons by relativistic electrons, while the second generally occurs under extreme laser conditions by photons colliding with the electromagnetic waves, for example, the laser fields. The first such an experiment was carried out by using the conventional paradigm at SLAC^19^. It is demonstrated that a 46 GeV linac-accelerated electron beam colliding with a 10^18^ W cm^−2^ laser is able to produce a few pairs (106±14), which shows a relatively weak QED effect.

State-of-art laser systems^20^ are capable of delivering a laser pulse with intensity up to 2 × 10^22^ W cm^−2^. The next-generation multi-PW lasers (for example, the XCELS and ELI facilities (the next generation of laser facilities, such as Exawatt Center for Extreme Light Studies (XCELS) and Extreme Light Infrastructure (ELI). Available at http://www.xcels.iapras.ru and http://www.eli-np.ro)) are expected to reach ∼10^24^ W cm^−2^ and beyond. This opens the door for studying light–matter interactions as well as QED effects in unexplored domains^1^,^4^,^21^,^22^. Diverse schemes have been proposed for energetic *e*^−^*e*^*+*^ pairs production via the BW process using ultrarelativistic lasers^23^,^24^,^25^,^26^,^27^,^28^,^29^,^30^,^31^. It is shown that using multiple colliding lasers^25^ for pair cascades in vacuum can reduce the required laser intensity down to ∼10^26^ W cm^−2^. This intensity is significantly smaller than the Schwinger value. An alternative scheme^26^,^27^ relies on the energetic electrons from a laser-driven gas jet or thin solid target by using either two counter-propagating lasers or a single laser. The positron beam produced is very bright and energetic. However, the required laser intensity is as high as ∼10^24^ W cm^−2^, still two orders of magnitude higher than that of the available lasers. Another challenge is the target transparence^27^ to the incident super intense lasers, which leads to the low efficiency of the BW process. By comparison, the laser-hohlraum scheme^28^ invokes the single-photon BW process with a much lower laser intensity but achieves a positron yield at the 10^5^ level only. More recently, it is proposed to combine the laser wakefield acceleration (LWFA^32^) with the positron generation by colliding the accelerated electron beam with a counter-propagating laser pulse^29^,^30^. The resulting positron yield can be up to ∼10^9^ (predicted by Blackburn *et al*.^29^), with a maximum density <10^20^ cm^−3^ (simulations by Lobet *et al*.^30^). This configuration allows for a compact linac, while the extraction and application of the produced positrons depend on additional laser and beam facilities, which is of significant importance for particle physics experiments, for example, a linear *e*^−^*e*^*+*^ collider. To date, an all-optical collider based on laser–plasma interactions for high-energy physics has yet to be realized.

For prolific pair creation via the BW process, high-energy and density γ photons are essential. The latter can be obtained by nonlinear Compton scattering^33^,^34^, bremsstrahlung radiation of electrons in a solid target^28^ or synchrotron radiation of electrons in a laser beam reflected from a thick foil^27^. Instead of using a solid or gas plasmas, here we present an efficient non-conventional scheme to generate extremely dense γ photons and copious numbers of *e*^−^*e*^*+*^ pairs by focusing two counter-propagating lasers at currently affordable laser intensity ∼10^22^ W cm^−2^ onto two near-critical-density (NCD) plasmas. The proposed scheme requires two steps. First, bright γ photons are produced by radiation-reaction trapped electrons in both NCD plasmas; second, the dense γ photons emitted from one NCD plasma collide with the focused counter-propagating laser in the other to initiate the multiple-photon BW process. We have carried out full three-dimensional (3D) particle-in-cell (PIC) simulations with collective QED effects incorporated. We demonstrate that the positron yield obtained is up to 1.05 × 10^11^, which is 10^6^-fold more than that obtained from the laser-hohlraum scheme^28^ and is two orders of magnitude larger than those by using the LWFA-accelerated electrons^29^,^30^. The peak positron density is as high as 4 × 10^22^ cm^−3^ with a cutoff energy of several GeV. This overdense *e*^−^*e*^*+*^ pair plasma source may find many practical applications and could serve as a compact linear collider with high luminosity.

## Results

### Overview of the scheme

When an electron absorbs multiple laser photons in the nonlinear Compton scattering process, it can radiate a high-energy photon. The radiated photons propagate through the laser fields and interact with the laser waves to produce *e*^−^*e*^*+*^ pairs via the multi-photon BW process. The probabilities of γ-photon emission and positron creation are determined by two relativistic and gauge invariant parameters^35^ (see Methods): 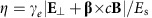 and 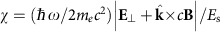, where **E**_⊥_ is the local electric field perpendicular to the electron velocity **β**, 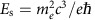 is the Schwinger electric field, and *ħk*(*ħω*) is the emitted photon momentum (energy). When a laser propagates parallel with an electron beam, it leads to *η*≅0, which is undesirable for high-energy γ photon emission and positron production; If the laser counter-propagates with the energetic electron beam, there is *η*≅1, which has been extensively investigated in past years^19^,^26^,^29^,^30^. Here we propose to use two lasers and two electron beams in an all-optical configuration realized simply by a pair of counter-propagating laser pulses in NCD plasmas. This enables one to have two sets of laser-electron beam colliding with *η*_1_≅1 and *η*_2_≅1 simultaneously (equivalent to a real *η* larger than 1), which could significantly enhance the γ photon emission and the pair production via the BW process.

### Radiation-reaction effect and radiation trapping of electrons

In extreme laser fields, the radiation damping force^36^,^37^,^38^ exerting on electrons could be expressed as 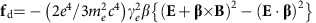, where *e* is the charge unit, *m*_e_ is the electron mass, and **β** is the normalized electron velocity by the light speed in vacuum *c*, **B** and **E** are the magnetic and electric fields. Here we keep only the main term proportional to 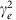 in the strong relativistic case. It is shown that the damping force **f**_*d*_ becomes significant enough to compensate for the Lorenz force **f**_*L*_=*q*(**E**+**β** × **B**), under laser intensity >10^22^ W cm^−2^, and it has to be taken into account in modelling laser–plasma interaction. As a result, the electron motion is profoundly altered. Instead of being scattered off transversely, electrons are trapped inside the laser field and perform extreme oscillations in the laser polarization direction. This is the radiation trapping effect^39^,^40^, which could lead to efficient synchrotron-like γ-ray emission. However, the simple test electron model^39^ suggests a threshold laser amplitude required to enter this regime, that is,


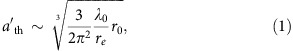


where *r*_0_ is the laser focal spot radius normalized by the wavelength *λ*_0_ and *r_e_*=*e*^2^/*m*_*e*_*c*^2^ is the classical electron radius. It is shown that the threshold is dependent on the laser focal size. In order to excite the multi-photon BW process with synchrotron-like γ rays, the threshold laser amplitude should meet 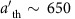, which is currently inaccessible. Therefore, in our scheme we first employ two cone-targets to focus the lasers. Instead of using a gas plasma or solid, we choose NCD plasmas filled inside the cones to increase the laser energy absorption and conversion so that more background electrons are provided and accelerated to enhance the γ-rays emission and positrons production.

**Figure 1:**
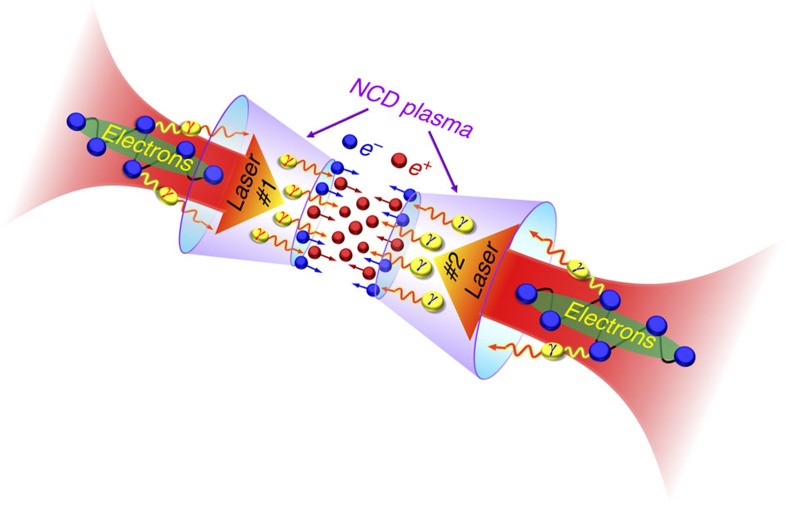
Extremely dense electron–positron pair production from near-critical-density plasmas. Two counter-propagating ultraintense laser pulses are focused from two directions onto the near-critical-density plasmas filled inside two cones (purple). The quivering electrons in the ultraintense laser fields experience large radiation-reaction forces by emitting photons so that a large number of electrons are trapped in the laser fields. These trapped electrons perform extreme oscillations in the transverse direction and emit bright γ rays (red- and blue–yellow) around the laser axis. Finally, copious numbers of *e*^−^*e*^*+*^ pairs are created via the multi-photon Breit–Wheeler process.

The scheme takes advantage of the radiation damping and trapping effect in the near-QED regime^39^,^40^. Figure 1 presents the schematic drawing of our basic configuration, where two counter-propagating laser pulses interact with the NCD plasmas inside a double-cone-targets. In this scheme, high-energy-density γ photons are emitted by the trapped energetic electrons in the NCD plasmas at the laser axis, which are accelerated by the intense laser fields. When the γ-photons collide with the focused counter-propagating laser waves from another direction, *e*^−^*e*^*+*^ pairs are efficiently produced via the multi-photon BW process. A positron beam produced in one NCD plasma can interact with the electron beam accelerated in the other NCD plasma, behaving like a microscopic *e*^−^*e*^*+*^ collider.

Here we demonstrate the feasibility of the scheme by using full 3D PIC code EPOCH with QED effects incorporated (see Methods). To benchmark the simulation results, we also perform a series of reference simulations using the QED-PIC code Virtual Laser–Plasma Lab. (VLPL^37^,^41^), which can reproduce the main results presented below.

### 3D PIC simulation results

**Figure 2:**
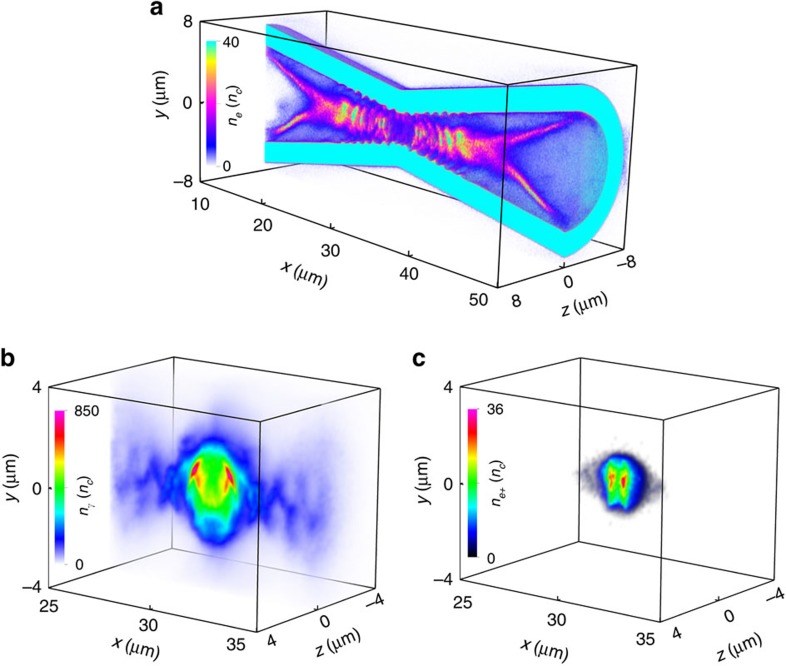
Three-dimensional particle-in-cell simulation results. Density distribution of electrons (**a**), γ photons (**b**) and positrons (**c**) at *t*=36*T*_0_. Both lasers enter the simulation box at *t*=0*T*_0_ and arrive at the open mouths of the double-cone-target at *t*=5*T*_0_. Two dense electron bunches are formed around the laser axis in the double-cone due to the radiation trapping effect, with high energy (∼5 GeV) and a high density (∼40 *n*_c_).

**Figure 3:**
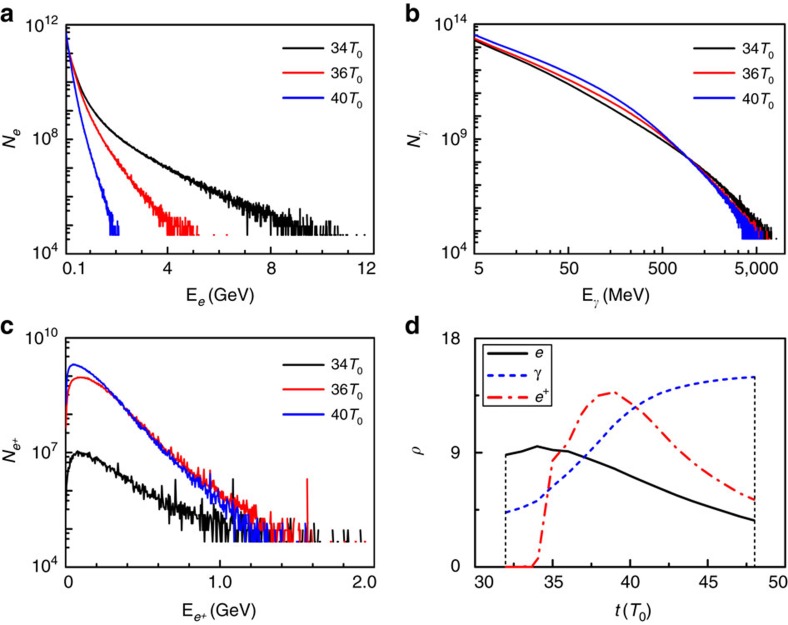
Evolution of the particle energy spectrum and the laser energy conversion efficiency. The energy spectra of electrons (**a**), γ-photons (**b**) and positrons (**c**) at *t*=34*T*_0_, 36*T*_0_ and 40*T*_0_. (**d**) The laser energy conversion to the trapped electrons *ρ*_*e*_(%), *γ*-photons *ρ_γ_*(%) and positrons *ρ*_*e*_+(0.01%), defined as the energy conversion efficiency *ρ*, as a function of the interaction time *t*.

Figure 2 illustrates the simulation results at *t*=36*T*_0_ (*T*_0_≈3.3 fs is the laser cycle), when both lasers overlap in the double-cone junction. It is shown that the laser intensity can be greatly boosted due to the coupling effect of nonlinear plasma effects and tightly focusing of the laser pulse in the cone^42^,^43^,^44^. The strengthened laser ponderomotive force accelerates the electrons both radially and forward with considerable radiation emitted. When the radiation damping effect is taken into account, electrons undergo a strong backward damping force. This force increases with the time and becomes comparable to the laser ponderomotive force. As a consequence, a large number of electrons are kicked back to the laser fields radially and accumulate near the laser axis, forming a dense electron bunch as shown in Fig. 2a. These electrons are ultrarelativistic with a cutoff energy of ∼5 GeV (Fig. 3a) and are well collimated around the laser axis with a peak density up to 40*n*_c_ (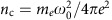 is the critical density). Additional simulations without the NCD plasmas and cone, respectively, indicate that the reduction of the laser threshold for the electron trapping is ultimately attributed to the nonlinear effect of the laser in the NCD plasmas-filled cone, which demonstrates the advantages of the cone structure over a plasma channel^40^. These trapped electrons travel almost along the laser-axis, inducing a strong poloidal self-generated magnetic field^40^,^43^. This results in additional pinching effect on the electrons. Therefore, the electron trapping or pinching near the laser axis originates from the radiation damping force and is remarkably enhanced by the magnetic pinching effect.

The trapped electrons co-move with the focused laser in the cone and keep oscillating with an amplitude of ∼2 μm in the laser field for a long time (Fig. 2a). During the process, two oxhorn-like electron bunches close to the cone mouths are also formed, resulting from the strong return currents in the cone. These trapped electrons emit a great deal of γ photons. At *t*=36*T*_0_, the photon density is up to 850*n*_c_ (Fig. 2b) and the cutoff energy is about several GeV (Fig. 3b). The corresponding average photon energy density is around 10^18^ J m^−3^, which is 10^7^ higher than the threshold for high-energy-density physics^45^. The production of such relativistic γ photons is crucial to studying the plasma dynamics and collective QED effects in laser–matter interactions^46^,^47^,^48^, which has many applications in diverse frontiers^1^,^21^,^23^, especially laboratory astrophysics.

The photon emission is mainly contributed by two processes: (1) the trapped electrons perform oscillations in the laser fields, like betatron oscillations in the bubble regime^32^,^49^,^50^; (2) the trapped high-energy electrons collide head-on with the opposite-propagating lasers, so that energetic photons are emitted by nonlinear Compton backscattering. Here the first process dominates the radiation over the second because the photon spectrum as seen in Fig. 3b is a typical synchrotron-like spectrum, while the scattered photons in the ultrahigh laser field limit via the second process would be only peaked at^51^ *ξ*/(1+*ξ*)*E*_*e*,max_≈1.5 GeV. Here the parameters are *ξ*=4*E*_*e*_*ħω*_0_/(*m*_*e*_*c*^2^)^2^≈0.18, *E*_*e*,max_≈10GeV at *t*=34*T*_0_, where *ħω*_0_≈1.2 eV is the laser photon energy. However, the second process enhances the high-energy γ-photon emission at later times (Supplementary Fig. 1; Supplementary Note 1). On the contrary, the photon emission by positrons created is a small fraction, since these positrons have a much smaller flux, energy, and density as compared with the trapped electrons in the laser fields.

These photons are distributed mainly around the laser axis with a cone angle *θ*_*γ*_∼1/*γ*_*e*_<1 mrad with respect to the cone axis in both cones. Later, they collide with the focused counter-propagating laser waves from the opposite directions, initiating the multi-photon BW process. Here, the BH process is intrinsically inefficient because of the low-Z NCD plasmas and the thin Al cone thickness^8^,^9^,^10^,^11^,^12^,^13^,^14^. Therefore, this process can be reasonably ignored in our simulations. Figure 2c presents the positron density distribution at *t*=36*T*_0_. A maximum positron density of ∼4 × 10^22^ cm^−3^ can be obtained with energies up to 1.6 GeV (Fig. 3c). This peak density is much higher than that reported in the both BW and BH experiments as well as relevant simulations^8^,^9^,^10^,^11^,^12^,^13^,^14^,^15^,^16^,^19^,^27^,^28^,^29^,^30^,^31^. The total positron yield is as high as 1.05 × 10^11^, which is more than an order of magnitude larger than that in laser foil interactions^27^, although our laser intensity is lower by more than an order of magnitude. As compared with the recent LWFA-aided scheme^29^,^30^, both the positron yield and density are two orders of magnitude higher.

Figure 3d presents the evolution of the laser energy conversion efficiency to the trapped electrons, γ photons, and positrons left in the simulation box. As the laser energy is soaked up and the electron energy grows, the damping process attenuates the laser wave and the laser energy is transferred to electrons and photons, and finally to positrons. At *t*=38*T*_0_, the positron energy approaches a maximum and then decreases by emitting photons in a similar way to electrons in the laser fields. The laser energy conversion efficiencies to the photons and positrons are peaked at 14.9% and 0.14%, respectively. With the same laser parameters, the efficiency of the positron production in our scheme is much higher than that of the LWFA-aided scheme^29^,^30^, making it very competitive as a compact positron source.

### Parametric influences and robustness of the scheme

**Figure 4:**
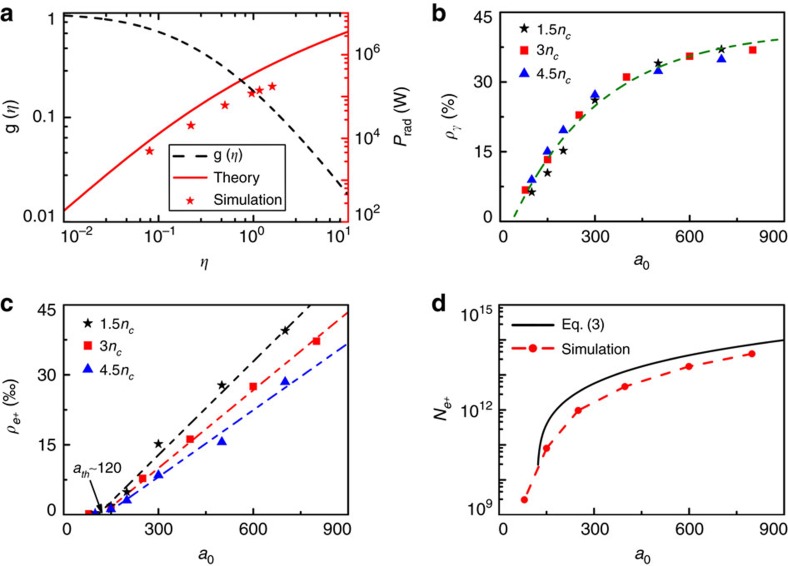
Results of theoretical predictions and numerical simulations. (**a**) The electron radiation power (red line) and the function *g*(*η*) (black dashed line) as a function of the parameter *η* in our scheme. The red asterisks represent the simulation results. The laser energy conversion efficiency to (**b**) the γ-photons and (**c**) positrons with different laser intensities and plasma densities. Here the green dashed line in **b** shows the fitted results. Note that there exists a laser threshold intensity in (**c**), *a*_th_∼120, for efficient positron production in our configuration. (**d**) The positron yield as a function of the laser intensity, based on the equation (3) and PIC simulations.

The robustness of the scheme is further demonstrated by using different laser intensities and NCD plasmas, as summarized in Fig. 4. Here the laser duration is changed to 8*T*_0_to save time, while other parameters are kept the same except for *a*_0_ and *n*_*e*_. As expected, both the photon emission and positrons creation are enhanced with the increase of the laser intensity. In the following, we compare our simulation results with theoretical predictions.

The quantum-corrected instantaneous radiation power by an electron is given by ref. 52, *P*_rad_=(4*πm*_*e*_*c*^3^/3*λ*_*C*_)*αη*^2^g(*η*)=*P*_*C*_g(*η*), where *λ*_*C*_ is the Compton wavelength, *α*=*e*^2^/*ħc*=1/137 is the fine-structure constant, *P*_*C*_=(4*πm*_*e*_*c*^3^/3*λ*_*C*_)*αη*^2^ is the classical power, and 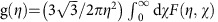 with *F*(*η*, *χ*) being the quantum-corrected synchrotron spectrum function as given by Erber^35^. Figure 4a shows the evolution of the radiation power. For comparison, we also give in Fig. 4a the simulation result calculated by collecting all γ photons' energy and then dividing this by the total number of trapped electrons. The radiation time is estimated to be of order of several laser cycles. We see that our simulation results agree well with the theoretical predictions, considering the fact that we neglect the low energy photons (<1 MeV) in the simulations. The numerical scaling of the laser energy conversion efficiency to the γ photons with different laser intensities and NCD plasmas is shown in Fig. 4b. By increasing the laser intensity, the laser energy conversion to the γ-photons increases at first and then saturates when the laser field amplitude *a*_0_>800. This can be attributed to the rapid annihilation of the high-energy γ-photons via the BW process. Note that the γ-photon emission is significantly limited by the number and energy of the trapped electrons.

In the simulations, we also observe a linear increase of the laser energy conversion to the positrons' kinetic energy, as illustrated in Fig. 4c. This tendency is valid for all considered NCD densities and laser intensities with *a*_0_>100. Qualitatively, the energy conversion efficiency can be approximately written as


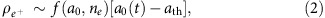


where *f*(*a*_0_,*n*_*e*_) is a factor dependent on the laser and NCD plasmas, and is a constant under a given initial condition, *a*_0_(*t*)=*a*_0_*g*(*t*), and *g*(*t*) is the temporal profile of the laser pulse. This implies there exists a threshold laser intensity or field amplitude, that is, *a*_th_∼120, for efficient pair creation in our configuration (Fig. 4c). We can understand the underlying physics simply in this way: when such a laser pulse is focused in the NCD plasmas-filled cone-target, its electric field amplitude can be increased by more than three times (depending on its focusing location in the cone), which has been confirmed by additional simulations using the same cone configuration as above. As a result, one obtains an enhanced laser amplitude, which approximates the equivalent theoretical laser threshold for the electron trapping in our cone-target, 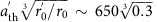, assuming a focusing spot radius of *r*_0_′≈0.3*r*_0_.

We finally compare the laser energy conversion efficiency in the simulations with theoretical predictions. Here the laser electric field can be increased up to 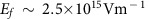 due to the enhanced pinching and focusing effect of the cone. The corresponding two critical parameters are given by *η*_*f*_∼3 and *χ*_*f*_∼2, which indicate effective excitation of both processes during the laser-NCD plasmas interaction. If we take *χ*=0.1 for example (see Methods), the required photon energy for pair creation is only 26 MeV, which is in reasonable agreement with the average energy of the γ-photons in our simulations. Considering *χ*_*f*_≳1 in our case, the characteristic positron energy is given by^53^ 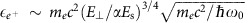, while the laser energy is 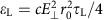. Thus we can estimate the final maximum positron yield by:


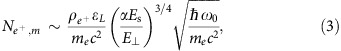


which is plotted in Fig. 4d. It is shown that our simulation results validate these theoretical estimations, especially for higher laser intensities. This further demonstrates the robustness of our scheme and validation of the simulations. If we scale our results to the upcoming lasers such as the XCELS (the next generation of laser facilities, such as Exawatt Center for Extreme Light Studies (XCELS) and Extreme Light Infrastructure (ELI). Available at http://www.xcels.iapras.ru and http://www.eli-np.ro), we can estimate the positron yield approaching ∼10^14^ with peak density of ∼10^25^ cm^−3^ and energy of tens GeV.

### Schematic of a possible experimental arrangement

**Figure 5:**
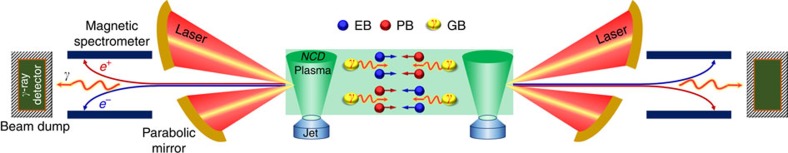
Schematic diagram of a possible experimental arrangement with strong lasers. Two counter-propagating 10 PW laser beams are focused by off axis parabolic mirrors on two gas or foam or cluster jets with near-critical-density, generating electron beams (EB), positron beams (PB) and γ-ray beams (GB). The focusing mirrors have small holes in the centre to extract the electrons (*e*^−^), positrons (*e*^*+*^) and γ-rays (γ), and to observe their interactions on axis.

A possible experimental arrangement of the scheme with two 10 PW ELI-NP laser beams is illustrated in Fig. 5. Instead of using a double-cone-targets, we can focus the two laser beams on two gas, foam or cluster jets to produce NCD plasmas^54^,^55^. Carbon-Nano-Tube foams^56^ can be also used for NCD plasma generation, which has been extensively applied in laser–plasma interactions. One can vary the gap between the two jets to optimize the γ-photon emission and pair production. The focusing mirrors have small holes on the interaction axis in order to separate the electrons, γ-photons, and positrons, and to diagnose their interaction dynamics on axis. The background radiation can be reduced by burying the gamma detectors into the electron beam-dump^23^, which is positioned on the axis of the two laser interaction, as schematically shown in Fig. 5.

The femtosecond synchronization^57^ of the two femtosecond laser pulses can be obtained because both pulses are split from the same pulse in our configuration (after the laser oscillator), travel nearly identical optical paths (in the laser amplifier chains) and the small temporal differences are compensated at the end. Indeed synchronization of ±50 fs has already been demonstrated experimentally with the two 0.5 PW laser beams of the Astra-Gemini Laser at STFC in the UK^57^ and the method described can be further improved. Because of the copious numbers of positrons and electrons expected, the measurement of the number and spectrum of electron–positron pairs and of γ-photons can be done in a single-laser-shot^12^,^23^, that is, there is no need to accumulate many shots as is typical in particle and nuclear physics experiments. The detectors could also be gated to the picosecond time-window of the laser shot in order to further increase the Signal-to-Noise ratio. Various interesting physics processes are likely to occur at the interaction area, including nonlinear Compton scattering, multi-photon BW process, *e*^−^*e*^*+*^ collider and γγ collider as discussed below.

## Discussion

Production of high-energy-density pair plasmas within a few tens of laser periods may open up new possibilities of studying astrophysical collective QED phenomena^1^,^21^ and high-energy particle physics^3^,^4^ in laboratories. Our scheme provides an efficient way to produce high-energy-density electrons and positrons, γ photons, and potentially other particles through their interactions, resulting in many applications. For example, this configuration is particularly suitable for applications as a non-conventional table-top *e*^−^*e*^*+*^ collider: the positron/electron beams and trapped energetic electron beams are generated in both NCD plasmas; when the electron beams in one NCD plasma collide head-on with the positron beams in the other NCD plasma (Supplementary Fig. 2; Supplementary Fig. 3; Supplementary Note 2), a compact *e*^−^*e*^*+*^ collider is expected, as indicated in Fig. 5. In the case of the future ELI facility (the next generation of laser facilities, such as Exawatt Center for Extreme Light Studies (XCELS) and Extreme Light Infrastructure (ELI). Available at http://www.xcels.iapras.ru and http://www.eli-np.ro; assume *I*_0_∼10^24^ W cm^−2^), the total positron number predicted is about 6 × 10^13^, with about 3 × 10^11^ positrons in the energy range between 2 and 2.5 GeV. This number is million times higher than detectable in current laser–plasma experiments so that the signal is strong enough to be detected in a single-laser-shot^12^,^23^. Assuming equal beams and Gaussian profiles in all dimensions with a beam size, conservatively, for example, 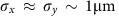, it is estimated that the peak geometric luminosity of such a 4−5 GeV centre-of-mass (CM) *e*^−^*e*^*+*^ collider is as high as 10^33^ cm^−2^ s^−1^, which is comparable to the state-of-art colliders worldwide^3^. One may even scale the proposed scheme to TeV CM *e*^−^*e*^*+*^ collision, which is a unique feature of our scheme as compared with the others^8^,^9^,^10^,^11^,^12^,^13^,^14^,^15^,^16^,^26^,^27^,^28^,^29^,^30^,^31^.

In our scheme, the low-emittance high-energy-density γ-photons in one NCD plasma can also collide with the other γ-photons from the second NCD plasma, which is a second γγ collider^51^ (Supplementary Fig. 1; Supplementary Note 1), an add-on to the *e*^−^*e*^*+*^ collider. Compared with the conventional linear colliders^3^, these new conceptual colliders based on laser–plasma interactions have many advantages, such as pure *e*^−^*e*^*+*^ collisions, low expense, compact size, and high luminosity, which may enable investigations in far-ranging scientific domains^4^,^21^,^58^ in future, for example, testing nonlinear phenomena such as mass-shift, spin-dependent effects, quantum gravity and so on.

In summary, we have presented a scheme on the generation of extremely dense *e*^−^*e*^*+*^ pairs via the multi-photon BW process at affordable laser intensity ∼10^22^ W cm^−2^ with the upcoming 10s PW lasers. In this scheme, bright γ rays are first produced by radiation-reaction trapped energetic electrons in the NCD plasmas. The photons then collide with the focused counter-propagating lasers to initiate the multi-photon BW process. A high-yield (1.05 × 10^11^) overdense (4 × 10^22^ cm^−3^) GeV positron beam is thus produced with a laser energy conversion efficiency as high as 0.14%. This highly energetic system may serve as a test bed for a variety of nonlinear QED physics and may be applied as a compact electron-position collider.

## Methods

### Two critical parameters in strong electromagnetic fields

The probability of photon emission and pair production can be written in terms of a differential optical depth^35^, 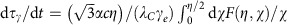 and d*τ*_±_/d*t*=(2*παc*/*λ*_*C*_)(*m*_*e*_*c*^2^/*ħω*)*χT*_±_(*χ*), respectively. Here *η* controls the photon emissivity via the quantum-corrected synchrotron function *F*(*η*,*χ*), and *χ* determines pair creation via the function 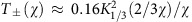. In our case, the two key parameters equals 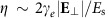 and 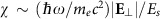, since the terms **β** × *c***B** and 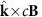 are parallel to the transverse laser field 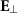. The Lorentz factor for electrons in the cone is assumed to be 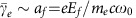, where *ħω*_0_ is the laser photon energy and *E*_*f*_ is the focused laser transverse electric field. Then, we obtain 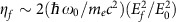, where *E*_0_=*m*_*e*_*cω*_0_/*e*. The characteristic photon energy can be described classically using the theory of synchrotron radiation as^26^ 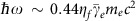. Thus the parameter *χ* is rewritten as 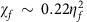. It is shown that, as *η* *≳*1 and *χ*≳1, the BW process dominates the positron production and quantum effects intervene significantly. Considering only *χ*≳0.1 in the photon–photon annihilation, the BW process also occurs, although it is relatively inefficient.

### Numerical modelling

The open-source PIC code EPOCH^27^,^59^ is used to perform the 3D simulations. The code has been equipped with the synchrotron radiation module, the radiation-reaction module, and the pair creation module (BW process), allowing self-consistent modelling of laser–plasmas interactions in the near-QED regime. In the code, the BW process is modelled by a probabilistic Monte Carlo algorithm^52^,^59^, which has been extensively applied recently. For simplicity, the *e*^−^*e*^*+*^ annihilation is ignored in the code.

In the simulations, two counter-propagating linearly polarized laser pulses are incident from the left and right boundaries of the box simultaneously, which have the same temporal-spatial profiles, that is, a transversely Gaussian distribution with *a*=*a*_0_exp(−*r*^2^/*r*_0_^2^) and a square temporal profile with a duration of *τ*_*L*_=12*T*_0_. Here the laser parameters are, respectively, *a*_0_=150, *r*_0_=5*λ*_0_, *T*_0_=3.3fs, *r*^2^=*y*^2^+*z*^2^, and *λ*_0_=1μm, which indicates a laser peak intensity of *I*_0_≈3 × 10^22^ W cm^−2^. Exposed in such a strong laser field, both electrons and protons can be pushed forward. The simulation box size is *x* × *y* × *z*=60*λ*_0_ × 16*λ*_0_ × 16*λ*_0_, sampled by cells of 3,000 × 240 × 240 with 27 macroparticles per cell. For simplicity, two symmetric aluminium cones are used to focus the incident laser pulses, both of which have a length of 50*λ*_0_ and a plasma density of *n*_0_=390*n*_c_. The left and right radius of each cone mouth are *R*=6 μm and *r*=1.5 μm, respectively. To enhance the laser energy absorption, the double-cone-targets are filled with NCD hydrogen plasmas, which has an initial density of *n*_*e*_=3*n*_c_. These parameters are tunable in simulations. For reference, we also compared the simulation results to the case with a Gaussian temporal pulse profile, which shows a comparable positron yield and density (Supplementary Fig. 4; Supplementary Fig. 5; Supplementary Note 3). Note that we only count the photons with energy >1 MeV in above simulations.

### Data availability

The data that support the findings of this study are available from the corresponding authors on request.

## Additional information

**How to cite this article**: Zhu, X.-L. *et al*. Dense GeV electron–positron pairs generated by lasers in near-critical-density plasmas. *Nat. Commun.* **7**, 13686 doi: 10.1038/ncomms13686 (2016).

**Publisher's note**: Springer Nature remains neutral with regard to jurisdictional claims in published maps and institutional affiliations.

## Supplementary Material

Supplementary Information Supplementary Figures 1-5, Supplementary Notes 1-3 and Supplementary References.
