# Supplementary material for: Dense GeV electron–positron pairs generated by lasers in near-critical-density plasmas
Source: Nat Commun. 2016 Dec 14;7:13686. doi: 10.1038/ncomms13686 (PMC5171869; doi:10.1038/ncomms13686)
Supplement: Supplementary Information — Supplementary Figures 1-5, Supplementary Notes 1-3 and Supplementary References. [file ncomms13686-s1.pdf]

Supplementary Figures:

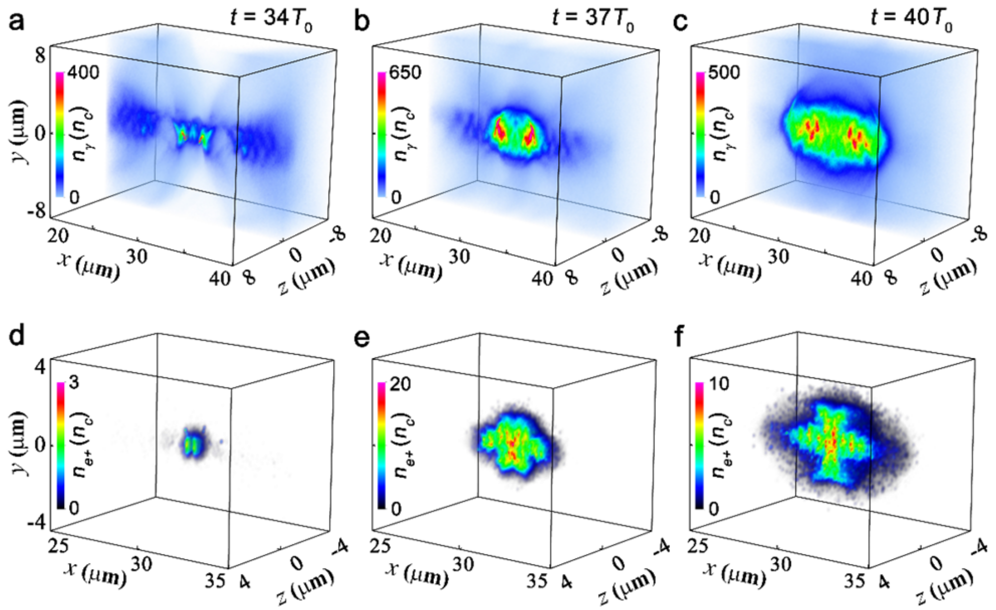

Supplementary Figure 1. Density evolution of γ-photons and positrons. Snapshots of the γ-photon and positron density distribution at $t = 34T_0$ (**a**, **d**), $t = 37T_0$ (**b**, **e**) and $t = 40T_0$ (**c**, f). Among them, (**a-c**) display the γ-photon density distributions and (**d-f**) present the positron density distributions.

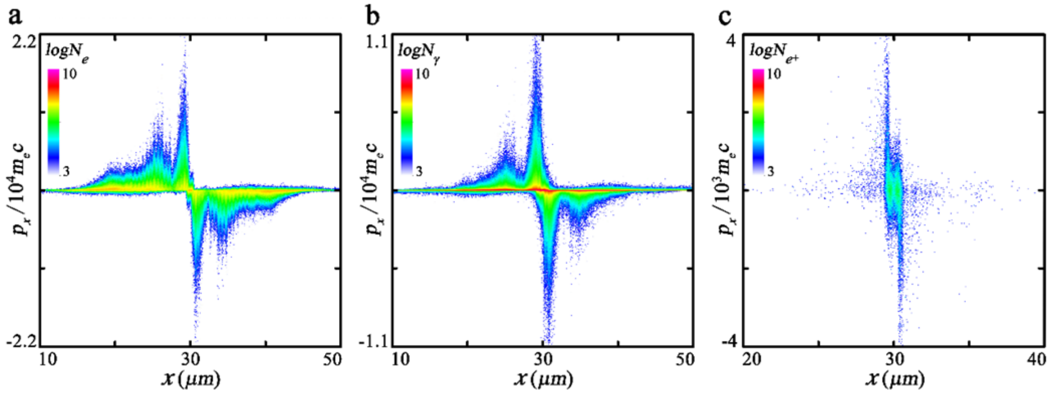

Supplementary Figure 2. Collision of counter-propagating beams. The distributions of (**a**) electrons, (**b**) γ-photons, and (**c**) positrons in phase space (x, p_x) at $t = 34T_0$. Here we only consider the zone $r_0 = 1\ \mu m$ where r_0 indicates the off-axis radius.

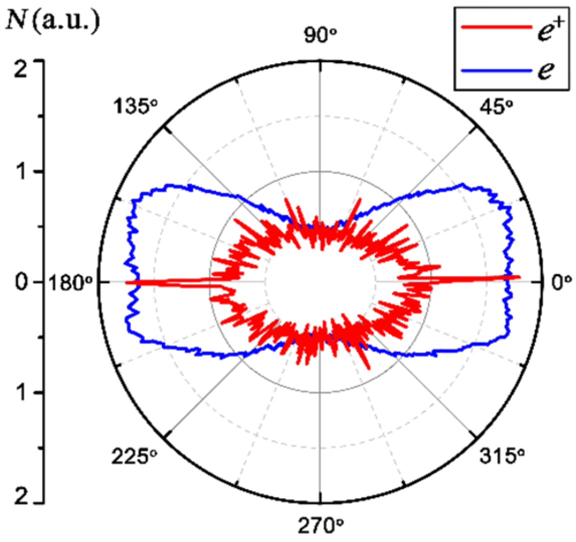

Supplementary Figure 3. The off-axis angular distributions of the trapped electrons and positrons. Here we only consider the zone $r_0 = 1\ \mu m$ where r_0 indicates the off-axis radius.

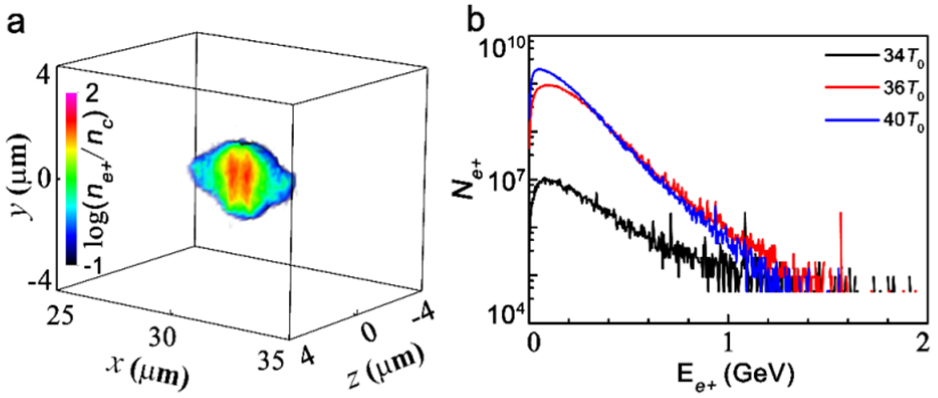

Supplementary Figure 4. The simulation results using lasers with a square temporal profile. (**a**) Density distribution of positrons. (**b**) Evolution of the positron energy spectrum.

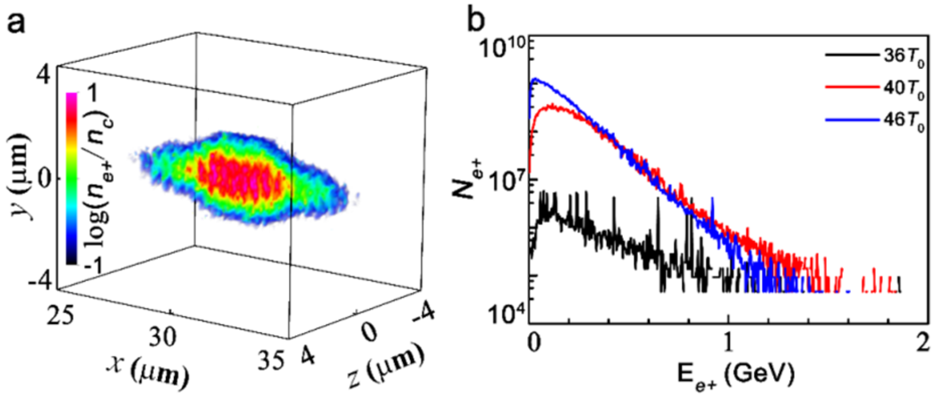

Supplementary Figure 5. The simulation results using lasers with a Gaussian temporal profile. (**a**) Density distribution of positrons. (**b**) Evolution of the positron energy spectrum.

Supplementary Note 1: Density evolution of γ-photons and positrons in 3D simulations

Detailed simulation parameters are given in Methods. Supplementary Figure 1a-c illuminates the γ-photon density evolution at $t = 34T_0$, $37T_0$, and $40T_0$. As expected, bright γ rays are emitted in both near-critical-density plasmas and the peak photon density increases from $400n_c$ at $t = 34T_0$ to $650n_c$ at $t = 37T_0$. A large number of electrons trapped in the laser fields oscillate and emit γ-photons, resulting in the decrease of the electron energy and increase of the low-energy electron flux, as illustrated in Fig. 3a in the main text. The lost energy is transferred to the photons and then positrons. At $t = 34T_0$, the nonlinear Compton backscattering becomes important and contributes to the γ-photon emission. Meanwhile, the Breit-Wheeler (BW) process is initiated and copious numbers of positrons are produced via the BW process. These positrons also oscillate in the laser fields and emit photons in a similar way to the electrons. This is the reason why we still observe an increase of the total photons in Fig. 3b in the main text, though the electron energy decreases significantly. These photons accumulate in the center, forming a hotspot with density up to $650n_c$ at $t = 37T_0$. Finally, the γ-photon yield is as high as $\sim 1.4 \times 10^{14}$.

The photons are mainly distributed in the laser axial direction and collide head-on with the counter-propagating laser waves to initiate the multi-photon BW process. Supplementary Figure 1d-f presents the positron density distribution at $t = 34T_0$, $37T_0$ and $40T_0$. As we can see, only a small amount of positrons are created at $t = 34T_0$, since the counter-propagating laser wave in the one cone is unapproachable for the γ-photons in the other cone so that their collision cross-section becomes very small at this time instant. The multi-photon BW process gets very efficient after $t = 34T_0$ when the high-energy-density γ-photons emitted collide with the counter-propagating laser waves.

Supplementary Note 2: The off-axis angular distributions of positrons

The head-on collision could be seen in the phase plots of the electrons/positrons, as shown in Supplementary Figure 2. At $t = 34T_0$, the beam #1, which has positive longitudinal momentum, collides head-on with the opposite-propagating beam #2 at $x = 30\mu m$. With time going on, the numbers of γ-photons and pairs increase, while the energy of electrons decreases significantly. The angular distributions of positrons and electrons are shown in Supplementary Figure 3. We can calculate the 'peak-emittance' by beam radius (μm)×beam divergence (radians). Here, the beam radius we quote is $r_0 = 1\,\mu m$ and the divergence we can take from Supplementary Figure 3 is about 1 degree for positron-spike and 30 degrees for positron bulk. Electron divergence is around 40 degrees. This would give peak-emittance at positron-angular-spike of 3.5×10^{-2} μm·radians, peak-emittance at positron-angular-bulk of

1.05 μm·radians, and peak-emittance at electron-angular-bulk of 1.40 μm·radians. The collisions are enhanced by the fact that all these electrons and positrons and γ-rays are at a very high density in a very small volume at the same time of a few hundred femtosecond[1-3]. This is very important for its application to a compact laser collider[1]. However, it is difficult to give an exact collision numbers because different reactions correspond to different cross section of collision. We estimate there are a very large number of millions of collisions in a single laser-shot[4]. And this would mean that we can get collision information in a single-laser-shot.

Supplementary Note 3: Additional simulations with a Gaussian-type laser temporal profile

In experiments, we usually use a Gaussian temporal laser profile. Here, we carried out additional simulations to compare the results to the square temporal laser pulse in the main text. In the simulations, the laser field amplitude has a temporal profile of $a_0(t) = a_0 g_{0,1}(t)$, where $g_0(t) = 1$ for the square temporal profile of the laser pulse and $g_1(t) = e^{-(t-\tau_0)^2/\tau_0^2}$ for the Gaussian temporal profile. Here, $\tau_0 = 12T_0$ and $a_0 = 150$ are used. The simulation results are shown in Supplementary Figure 4 and Supplementary Figure 5. It is obvious to see that the magnitudes of positron energy, density, and energy spectrum in both cases are all at similar levels.

We can also evaluate the impact of the laser temporal profile analytically. From Equation 2 in the main text, we can vary the laser temporal profile by the function $g(t)$. From equations (2) and (3) in the manuscript, we can get the ratio of the positron yield by a laser with a Gaussian temporal profile to that with a square one as follows:

$$\mathrm{R}_{N_{e^+}} = \frac{N_{e^+,Gauss}}{N_{e^+,square}} \sim \frac{1}{\tau_0(a_0 - a_{th})} \int_{t_1}^{t_2} [a_0 g_1(t) - a_{th}] g_1(t) dt. \quad (1)$$

Considering the fact that only the laser with a temporal intensity large than the threshold value (a_{th}~120) can efficiently produce positrons (see Fig.4(c) in the main text), we can estimate the positron production in the Gaussian laser temporal case easily. For the laser pulse with $a_0 = 150$, we find $\mathrm{R}_{N_{e^+}}$~0.59. By comparison, the total positron yield in simulations is $\sim 6 \times 10^{10}$, which is approximately 0.57 times that in the square laser case. This indicates the validation of our estimation and the robustness of the scheme. Therefore, a Gaussian temporal profile of laser pulse does not significantly change the positron production in our configuration.

Supplementary References

1. Di Piazza, A., Müller, C., Hatsagortsyan, K. Z. & Keitel, C. H. Extremely high-intensity laser interactions with fundamental quantum systems. *Rev. Mod. Phys.* **84**, 1177-1288 (2012).

2. Ridgers, C. P. *et al.* Dense Electron-Positron Plasmas and Ultraintense γ rays from Laser-Irradiated Solids. *Phys. Rev. Lett.* **108**, 165006 (2012).

3. Luo, W. *et al.* Dense electron-positron plasmas and gamma-ray bursts generation by counter-propagating quantum electrodynamics-strong laser interaction with solid targets. *Phys. Plasmas* **22**, 063112 (2015).

4. Zhu, K., Yuan, C. Z. & Ping, R. G. Cross sections of $e^+e^-\to\gamma VV$ and $e^+e^-\to\gamma\gamma V$. *Phys. Rev. D* **78**, 036004 (2008).
